# Inactive bowel movement and stroke are associated with increased risks of mild cognitive impairment among community-living Singapore elderly

**DOI:** 10.18632/aging.103674

**Published:** 2020-09-09

**Authors:** Kai-Yong Huang, Xian-Yan Tang, Li Yang, Zhi-Yong Zhang, Kaisy Xinhong Ye, Qing-Feng Shen, Xiu Wang, Xiang-Hua Zhu, Xiao-Wei Huang, Guo-Dong Lu, Lei Feng

**Affiliations:** 1 Department of Occupational and Environmental Health, School of Public Health, Guangxi Medical University, Nanning, Guangxi Province, P.R. China; 2 Department of Epidemiology and Biostatistics, School of Public Health, Guangxi Medical University, Nanning, Guangxi Province, P.R. China; 3 School of Public Health, Guilin Medical University, Guilin, Guangxi Province, P.R. China; 4 Department of Psychological Medicine, Yong Loo Lin School of Medicine, National University of Singapore, Singapore; 5 Xuzhou Oriental People's Hospital, Xuzhou, Jiangsu Province, P.R. China; 6 Department of Neurology, Beijing Chuiyangliu Hospital, Beijing, P.R. China; 7 Department of Toxicology, School of Public Health, Guangxi Medical University, Nanning, Guangxi Province, P.R. China; 8 The Guangxi Universities Key Laboratory of Prevention and Control of Highly Prevalent Disease, School of Public Health, Guangxi Medical University, Nanning, Guangxi Province, P.R. China; 9 Centre for Healthy Longevity, National University Health System, Singapore

**Keywords:** mild cognitive impairment, bowel movement, fruit consumption, dietary habits, stroke

## Abstract

Mild cognitive impairment (MCI), as a preclinical phase of dementia, provides an invaluable time window for intervention. Besides several proposed modifiable risk factors, the associations of MCI with dietary habits and bowel movement are not well clarified. We thus conducted a cross-sectional study of community-living Singapore elderly and focused on the relationship of clinically diagnosed MCI with dietary habits and bowel movement frequencies. The multiple logistic regression results showed that frequent (≥4 days per week) fruit consumption (*P* = 0.004), active (≥4 days per week) bowel movement within 10 minutes (*P* = 0.027), and years of schooling were negatively associated with MCI occurrence. In contrast, medical comorbidities including hypertension, stroke, and cataract/glaucoma were found to be risk factors. Furthermore, a Bayesian network model of causal inference detected five hypothesized causal-association paths leading to MCI, namely bowel movement, stroke, years of schooling via fruit consumption, hypertension via stroke and hypertension via cataract/glaucoma. The combination of the two direct factors (inactive bowel movement and stroke) reached a maximum conditional probability of 60.00% for MCI occurrence. Taken together, this study was the first to link bowel movement with MCI occurrence. In addition, it suggested five modifiable hypothesized causal-association paths to MCI.

## INTRODUCTION

Dementia, which occurs mainly in people older than 65 years, is one of the greatest global challenges for health and social care in the 21^st^ century [1]. Fifty million people worldwide lived with dementia in 2018, and the total number may triple to 152 million by 2050 [2]. The annual global cost of dementia treatment and care has reached one trillion US dollars and will double by 2030 [2]. Unfortunately, there is no effective disease-modifying pharmaceutical treatment available to cure or prevent dementia. Mild cognitive impairment (MCI) is a transitional state between the cognitive changes of normal aging and dementia [3], with a prevalence varying from 4% to 19% among people aged 60 and above [1]. MCI is also regarded as a preclinical phase of dementia, because around 22% of MCI-affected people would progress to dementia within 3 to 10 years upon diagnosis of MCI, while only 3% of non-MCI people develop dementia [4]. This pre-disease state of MCI opens an invaluable therapeutic time window to intervene or prevent further deterioration into dementia.

It was estimated that around one third of dementia burdens could be prevented theoretically by controlling modifiable risk factors [1]. Preventive countermeasures are composed of two major categories: management of medical comorbidities and modifications of lifestyles. First, high-risk medical comorbidities (e.g. midlife hypertension, obesity and hearing loss; late-life depression and diabetes) account for 17% of risks to develop dementia. Active treatments of these medical comorbidities were recommended to control MCI and prevent deterioration into dementia. Second, adopting healthy lifestyles (e.g. active engagements of social and physical activities, cognitive stimulation activities and smoking cessation) may control another 10% risks. Unfortunately, dietary factors were not included in the model, and their contributions are not calculated [1], although adherence to a Mediterranean diet was suggested to be beneficial for controlling vascular and metabolic risk factors. Previous studies have reported that adequate or excess intakes of fruit and/or vegetables were associated with a reduced risk of cognitive impairments [5]. Numerous preclinical studies have found that polyphenolic antioxidants found in fruits and vegetables could effectively decrease and even block neuronal death and thereby reduce the occurrence of dementia, especially in cell studies and in animal models [6].

Another close but neglected connection is bowel movement, which is affected by individuals’ dietary habits and the stool contents. Inactive bowel movement and constipation are common gastrointestinal symptoms (e.g., stool hardness, a prolonged and difficult passage of stool, or a feeling of unfinished evacuation) [7]. It has been proposed that gut-brain axis dysfunction may play a role in neurological diseases, strengthening the importance of gut flora in a biochemical signaling cascade between the gut and central nervous system. Constipation could precede clinical motor symptoms, particularly Parkinson’s disease (PD), by many years [8]. Based on a large-scale prospective cohort study (the Honolulu-Asia Aging Study) with 12 years’ follow-up, Abbott and colleagues made a landmark finding that infrequent bowel movement was correlated with an increased risk of PD [9]. A case-control study by Savica et al. using medical records documentation in the Rochester Epidemiology Project later reiterated the positive association between constipation (which was recorded 20 years or even more before the onset of motor impairments) and an increased risk of PD development. However, it is still unclear whether constipation may affect cognitive functions apart from motor neuron diseases. A few studies [10] have pointed out that patients affected by dementia with Lewy bodies had a higher prevalence of constipation. The authors suggested including examinations of constipation together with other non-cognitive symptoms, e.g., hyposmia and sleep behavior disorders, for the prodromal diagnosis of dementia.

In the present study, we aimed to dissect the associations of bowel movement frequencies and dietary habits with clinical-diagnosed MCI in a population-based cross-sectional study [the Diet and Healthy Aging (DaHA) study] in Singapore. For this observational study, we applied multiple logistic regression analyses to determine the adjusted odds ratios (AOR) of bowel movement on MCI occurrence. A Bayesian network approach that integrates causal diagram model and probabilistic model was then applied to infer the causal relationships between exposure variables (bowel movement frequencies and other possible influencers) and outcome variable (MCI occurrence). Qualitatively, the conceptual framework of causal association and the hypothesized causal-association paths among exposure variables were detected via a direct acyclic graph (DAG). Quantitatively, the magnitudes of causal association were inferred via the conditional probability table of the outcome variable under the combinations of direct factors. Moreover, the predictive reasoning forwarded from exposure variables and the importance scores of exposure variables were determined with a Bayesian network. This methodology has been conducted successfully in the field of neurological diseases. Kuipers and colleagues inferred the causal links between psychotic and neurotic symptoms in the British general population with directed acyclic graphs in a dynamic Bayesian network approach [11]. Rembach *et al.* employed the same approach to reveal the complex biological interactions specific to Alzheimer’s disease (AD) and assessed the biological connections among AD-associated serum protein biomarkers [12].

## RESULTS

### Socio-demographic characteristics and medical comorbidities of the study population

Among the enrolled participants (*N*=751), 119 elders (15.9%) were clinically diagnosed with MCI as described in the Methods section, while the rest were defined as non-MCI elders (or cognitive-healthy ageing control). MCI elders (aged 68.9±6.9 years) were 1.5 years older on average than the non-MCI participants (67.4±5.3, *P*=0.007, Table 1), but had markedly less education (4.8±4.5 *vs.* 6.8±4.1 years of schooling, *P*=0.003). There were no differences between these two groups in gender, marital status, or smoking and drinking habits. On the other hand, MCI patients differed from the non-MCI elders in some medical comorbidities, including hypertension (63.9% *vs.* 46.0%, *P*<0.001), hyperlipidemia (63.0% *vs.* 50.8%, *P*=0.014), diabetes mellitus (25.2% *vs.* 16.1%, *P*=0.017), stroke (8.4% *vs.* 2.5%, *P*=0.001), and cataract/glaucoma (48.7% *vs.* 32.3%, *P*=0.001).

**Table 1:** Socio-demographic characteristics and medical comorbidities comparisons between MCI elders and non-MCI elders.

**Variables**	**non-MCI n (%)**	**MCI n (%)**	**F or *χ^2^***	**P-value**
**Age** (Mean ± SD)	67.4 ± 5.3	68.9 ± 6.9	7.426	**0.007**
**Years of schooling** (Mean ± SD)	6.8±4.1	4.8±4.5	8.646	**0.003**
Gender*			0.924	0.336
Male	197 (31.3)	32 (26.9)		
Female	432 (68.7)	87 (73.1)		
Marital status*			2.253	0.133
Married	455 (72.3)	78 (65.5)		
Divorced, widowed or single	174 (27.7)	41 (34.5)		
**Smoking**			0.088	0.767
< 1 per week	594 (94.0)	111 (93.3)		
≥1 per week	38 (6.0)	8 (6.7)		
**Drinking**			0.342	0.559
< 1 per week	598 (94.6)	111 (93.3)		
≥1 per week	34 (5.4)	8 (6.7)		
**Hypertension**			12.729	**< 0.001**
Yes	291 (46.0)	76 (63.9)		
No	341 (54.0)	43 (36.1)		
**Hyperlipidemia**			6.013	**0.014**
Yes	321 (50.8)	75 (63.0)		
No	311 (49.2)	44 (37.0)		
**Diabetes** **mellitus**			5.688	**0.017**
Yes	102 (16.1)	30 (25.2)		
No	530 (83.9)	89 (74.8)		
**Stroke**			10.331	**0.001**
Yes	16 (2.5)	10 (8.4)		
No	616 (97.5)	109 (81.6)		
**Heart attack**			1.556	0.212
Yes	22 (3.5)	7 (5.9)		
No	610 (96.5)	112 (94.1)		
**Irregular heart beat**			0.601	0.438
Yes	27 (4.3)	7 (5.9)		
No	605 (95.7)	112 (94.1)		
**Heart failure**			0.046	0.831
Yes	9 (1.4)	2 (1.7)		
No	623 (98.6)	117 (98.3)		
**Cataracts/glaucoma**			11.946	**0.001**
Yes	204 (32.3)	58 (48.7)		
No	428 (67.7)	61 (51.3)		
**Gastrointestinal problems**			0.921	0.337
Yes	52 (8.2)	13 (10.9)		
No	580 (91.8)	106 (89.1)		
**Anxiety disorder**			3.627	0.057
Yes	5 (0.8)	4 (3.4)		
No	627 (91.2)	115 (96.6)		

Note: Continuous variables are expressed by mean±SD. * Due to the missing values in some variables, the total numbers may not equal to 632 or 119. Abbreviations: MCI, mild cognitive impairment; SD, standard deviation.

### Comparisons of bowel movement frequencies and dietary habits between MCI elders and non-MCI elders

In order to determine the association of bowel movement frequencies and dietary habits with MCI occurrence, we collected the self-reported frequencies of these factors (e.g., “How often per week do you have a bowel movement within 10 minutes?”) in the studied population. We compared the proportions of bowel movement frequencies and different dietary habits between MCI patients and non-MCI elders. As shown in Table 2, a higher proportion of MCI elders complained infrequent (≤3 days per week) bowel movement than the non-MCI elders (26.7% *vs.* 17.2%, *P* = 0.013).

**Table 2:** Bowel movement and dietary habits variables comparisons between MCI elders and non-MCI elders.

**Variables**	**non-MCI n (%)**	**MCI n (%)**	***χ^2^***	**P-value**
**Have a bowel movement within 10 minutes**			6.107	**0.013**
≤3 per week	109 (17.2)	32 (26.7)		
≥4 per week	523 (82.8)	87 (73.3)		
**How often consume meat and meat product**			6.074	**0.014**
≤3 per week	326 (51.6)	76 (63.9)		
≥4 per week	306 (48.4)	43 (36.1)		
**How often consume milk and dairy product**			0.001	0.972
≤3 per week	341 (54.0)	64 (53.8)		
≥4 per week	291 (46.0)	55 (46.2)		
**How often consume green vegetables**			1.040	0.308
≤3 per week	65 (10.3)	16 (13.4)		
≥4 per week	567 (89.7)	103 (86.6)		
**How often consume fruits**			15.740	**< 0.001**
≤3 per week	120 (19.0)	42 (35.3)		
≥4 per week	512 (81.0)	77 (64.7)		
**How often consume legumes**			3.371	0.066
≤3 per week	539 (85.3)	109 (91.6)		
≥4 per week	93 (4.7)	10 (8.4)		
**How often consume eggs**			4.238	**0.040**
≤3 per week	520 (82.3)	107 (90.0)		
≥4 per week	112 (17.7)	12 (10.0)		
How often consume sea fish*			0.021	0.884
≤3 per week	327 (52.0)	61 (51.3)		
≥4 per week	302 (48.0)	58 (48.7)		
**How often consume seaweed #**				0.497
≤3 per week	616 (97.5)	118 (99.2)		
≥4 per week	16 (2.5)	1 (0.8)		
How often consume tofu *			2.549	0.110
≤3 per week	537 (85.2)	108 (90.8)		
≥4 per week	93 (14.8)	11 (9.2)		
How often consume carrot*			0.657	0.417
≤3 per week	476 (75.6)	85 (72.0)		
≥4 per week	154 (24.4)	33 (28.0)		
How often consume celery*			1.011	0.315
≤3 per week	590 (93.8)	115 (96.6)		
≥4 per week	39 (6.2)	4 (3.4)		
**How often consume walnut**			1.745	0.187
≤3 per week	587 (92.9)	115 (96.6)		
≥4 per week	45 (7.1)	4 (3.4)		
**How often consume coffee**			3.352	0.067
≤3 per week	202 (32.0)	28 (23.5)		
≥4 per week	430 (68.0)	91 (76.5)		

Note: * Due to the missing values in some variables, the total numbers may not equal to 632 or 119. # Analyzed by Fisher's Exact Test. Abbreviations: MCI, mild cognitive impairment.

Dietary habits were also associated with MCI occurrences. The univariate analyses found that a higher proportion of MCI elders ate fruits infrequently (≤3 days per week) than their cognitive healthy peers (35.3% *vs.* 19.0%, *P*<0.001). Similar trends were observed for meat (63.9% *vs*. 51.6%, *P* = 0.014) and egg consumption (90.0% *vs.* 82.3%, *P* = 0.040). In contrast, there were no statistical differences in their consumptions of milk and dairy products, vegetables (including green vegetables, celery, and carrots), tofu, sea products (including sea fish and seaweed), or walnut between subgroups with or without MCI. The infrequent legume consumption (91.6% *vs.* 85.3%, *P* = 0.066) and coffee consumption (23.5% *vs.* 32.0%, *P* = 0.067) were slightly different between the two groups but failed to reach the threshold of statistical difference.

Because individuals’ dietary habits may affect stool contents and bowel movements, we then determined whether there were any correlations between bowel movement frequencies and dietary habits. The results (Supplementary Table 1) showed that only fruit consumption but not the other dietary habits were associated with bowel movement. The elders who had active bowel movement consumed fruit in a higher frequency (≥4 days per week, 79.8%) than those who had inactive bowel movement (71.6%, *P* = 0.033).

### Multiple logistic regression analyses confirmed the independent associations of bowel movements and fruit consumption with MCI

To further determine the independent associations of bowel movements and dietary habits with MCI occurrence and adjust for the influences of other factors (e.g., age, education, medical comorbidities), we performed multiple logistic regression analyses. Those factors with statistical differences *P* < 0.1 in the univariate *chi*-square or analysis of variance (ANOVA) tests (Tables 1 and 2) were included in a multiple logistic regression model (Table 3). It is worth noting that active bowel movement (≥ 4 days per week) was associated with decreased risk of MCI occurrence [adjusted odds ratios (AOR) = 0.582, 95% confidence interval (CI) = 0.360-0.941, *P* = 0.027]. Similarly, years of schooling (AOR = 0.906, 95%CI = 0.861-0.954, *P* < 0.001) and active fruit consumption (AOR = 0.516, 95% CI = 0.330-0.808, *P* = 0.004) were found to be associated with decreased risk of MCI occurrence. In contrast, medical comorbidities including hypertension (AOR = 1.770, 95%CI = 1.159-2.705, *P* = 0.008), stroke (AOR = 2.514, 95%CI = 1.065-5.936, *P* = 0.035), and cataracts/glaucoma (AOR = 1.803, 95%CI = 1.190-2.731, *P* = 0.005) were statistically associated with increased risks of MCI.

**Table 3:** Multiple logistic regression analysis for MCI.

**Variables**	***β***	**Wald**	**P-value**	**AOR**	**95% CI**
Years of schooling	-0.099	14.191	< 0.001	0.906	0.861-0.954
Hypertension	0.571	6.981	0.008	1.770	1.159-2.705
Stroke	0.922	4.423	0.035	2.514	1.065-5.936
Cataracts/glaucoma	0.589	7.740	0.005	1.803	1.190-2.731
Have a bowel movement within 10 minutes (≥4 days per week)	-0.541	4.886	0.027	0.582	0.360-0.941
Consume fruits (≥4 times per week)	-0.662	8.374	0.004	0.516	0.330-0.808

Note: Model was adjusted for gender, age, marital status, smoking, drinking. Abbreviations: AOR, adjusted odds ratio; CI, confidence interval.

### Bayesian network model of the influencing factors of MCI

**Figure 1:**
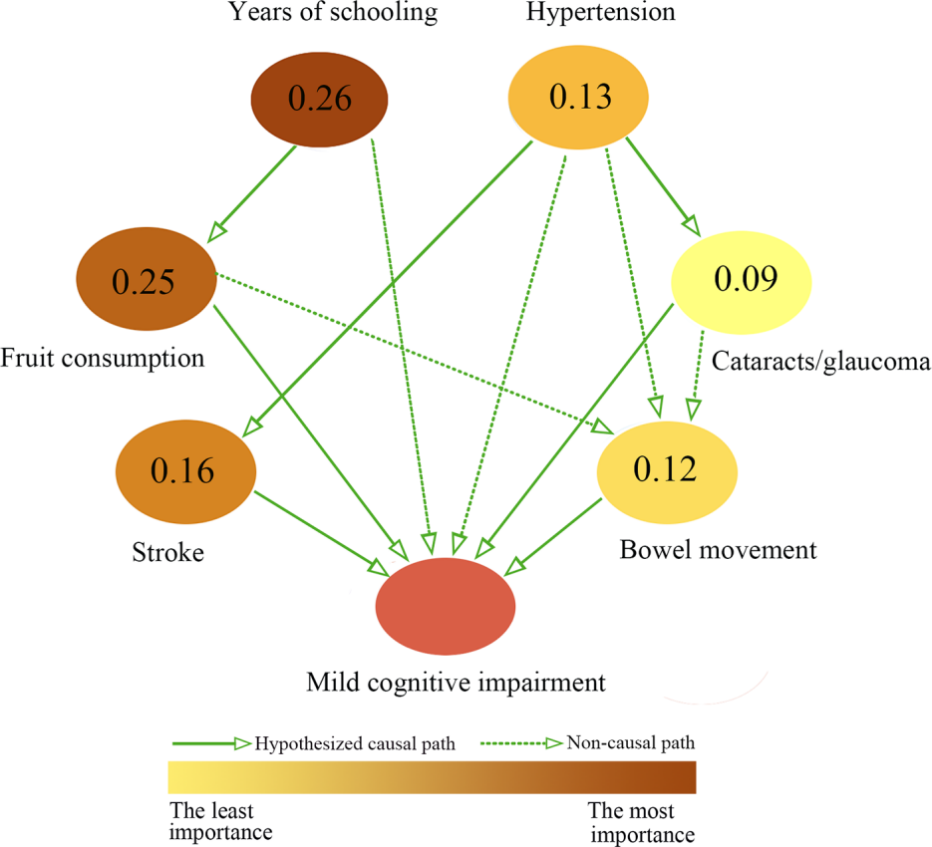
**Causal inference of MCI by Bayesian network analysis.** Bayesian network approach detected the hypothesized causal-association diagram between MCI and exposure variables, and the importance of exposure variables. The magnitude of values presented at each node denote the importance of exposure variables. In other words, the higher magnitude the value is, more important the variable. Meanwhile, the importance of variables was visualized via the gradual changes of color of nodes.

The hypothesized causal-association paths between MCI occurrence and significant influencers found in the multiple logistic regression analyses were explored by the Bayesian network model of causal inference. As shown in Figure 1, the results indicate five hypothesized causal-association paths leading to MCI occurrence. Among these, both bowel movement and stroke were directly associated with MCI occurrence. Years of schooling might be indirectly associated with MCI through fruit consumption. In addition, hypertension could be indirectly associated with MCI through either stroke or cataracts/glaucoma.

Other information obtained from the Bayesian network model of causal inference was the importance of factors. Years of schooling was the most important factor, with an importance score of 0.26, followed by fruit consumption (0.25), stroke (0.16), hypertension (0.13), bowel movement (0.12) and cataract/glaucoma (0.09).

Finally, the inference probabilities of Bayesian network forwarding from the two direct factors (inactive bowel movement and stroke) to MCI were analyzed. Specifically, the combination of inactive bowel movement (≤ 3 days per week) and stroke had a maximum conditional probability of 60.00% (Table 4). But inactive bowel movement alone and stroke alone had lower probabilities of 44.51% and 29.54%, respectively. Furthermore, the combination of active bowel movement and no-stroke medical history had the least probabilities of 12.92%.

**Table 4:** Probability inference of Bayesian network for the direct factors of MCI.

**Paternal nodes**		**MCI**
**Have a bowel movement within 10 minutes**	**Stroke**	**Yes (%)**
≤3 days per week	Yes	60.00
≤3 days per week	No	44.51
≥4 days per week	Yes	29.54
≥4 days per week	No	12.92

Abbreviations: MCI, mild cognitive impairment.

## DISCUSSION

To the best of our knowledge, the present study was the first to examine the association between bowel movement and MCI. We found that active bowel movement, fruit consumption, and years of schooling were associated with decreased risk of MCI, while medical comorbidities including hypertension, stroke, and cataracts/glaucoma were unfavorable risk factors. The Bayesian network model further revealed that five hypothesized casual-association paths were important for MCI occurrence. Among them, inactive bowel movement and stroke were directly connected with MCI occurrence.

Gut-brain axis dysfunction has been hypothesized to be an essential factor in neurological diseases. Alterations of gut microbiota may mechanically promote neurological inflammation, insulin resistance [7, 13], and consequent neurological pathogenesis [14]. As a common digestive symptom, constipation (or inactive bowel movement) was well documented to increase the risk of motor neurological diseases. In addition to some case-control studies, the prospective Honolulu-Asia Aging Study [9] convincingly demonstrated that people with inactive bowel movement had increased risk for developing PD. However, there is a scarcity of solid epidemiological evidence associating bowel movement (or constipation) with cognitive impairments. Some observational studies have suggested a possible role of bowel movement in MCI or even dementia. On one hand, a few postmortem reports observed higher occurrences of dementia-related pathological changes (e.g., the presence of Lewy bodies and a decrease of substantia nigra neurons) in the brains of those who had been affected by constipation [15, 16]. On the other hand, some studies also reported that about 20% of dementia patients had constipation symptoms [17], which is consistent with findings of the present study that 26.7% MCI-affected elders had inactive bowel movement (Table 2). MCI-affected patients also observed gut microbiota alterations [18]. More importantly, these constipation symptoms can precede the onset of memory impairment by years.

The current study might support the gut-brain axis hypothesis by demonstrating that bowel movement was independently associated with MCI occurrence after adjusting for other influencing factors (Table 3). Furthermore, both bowel movement and stroke were directly associated with MCI in the causal diagram model of Bayesian network. The combination of inactive bowel movement and stroke increased the conditional probability to 60.00%, which was higher than inactive bowel movement alone or stroke alone (Table 4). Very recently, GV-971 (a sodium oligomannate) was found to be effective in relieving neuroinflammation and preventing cognitive impairments in a mouse AD model [19]. The authors concluded that the inhibitory actions of GV-971 on gut dysbiosis were responsible for the observed neuroprotective effects. Some promising results of a phase-III randomized clinical trial using GV-971 to treat mild dementia patients were presented in a conference, but the full results have not been published. It is worth noting that GV-971 was given conditional approval to treat patients with dementia by the China Food and Drug Administration in November 2019. Whether this drug could prove long-term efficacy in multi-regional randomized clinical trials and whether the proposed gut-brain axis dysfunction theory is valid warrant further studies.

The Bayesian network model indicated that education and fruit consumption were two important upstream factors influencing MCI, and they have the highest importance scores at 0.26 and 0.25 respectively (Figure 1). These two factors formed one hypothesized causal path directing from education to fruit consumption and then to MCI. This result is in line with a previous study observing that elderly with high education levels had higher fruit intake [20]. It is well established that childhood education enables individuals to increase cognitive reserve and even maintain cognitive function when they grow old. Education beyond primary school is regarded as the second important modifiable beneficial factor for dementia [1]. In the present study, around 42% of resident elders had no more than 5 years of education. Our results consistently showed that education (years of schooling) was negatively associated with MCI occurrence (Table 3). On the other hand, frequent consumption of fruit (≥4 days per week) was also negatively associated with MCI occurrence (AOR = 0.516, 95% = CI 0.330-0.808, *P* = 0.004) in our study (Table 3). Because education and fruit consumption belong to the same hypothesized causal path, the combination of poor education (≤ 5 years of schooling) and infrequent fruit consumption (≤3 times per week) reached a conditional probability at 32.86% (Supplementary Table 2), which is far less than the combination of the two direct factors (inactive bowel movement and stroke) with a conditional probability at 60.0% (Table 4).

Besides bowel movement and education via fruit consumption, the other three hypothesized causal-association paths directing to MCI were stroke, hypertension via stroke, and hypertension via cataracts/glaucoma, according to the Bayesian network model of causal inference (Figure 1). These medical comorbidities were statistically associated with MCI occurrence in the multiple logistic regression analyses (Table 3). Stroke and hypertension are well-known risk factors for MCI and dementia [1, 21, 22]. Active treatment of hypertension, however, significantly reduced the risk of stroke and MCI [23]. In addition, hypertension increases the risk of cataracts and/or glaucoma in population studies and rat experimental models [24]. Cataracts and glaucoma caused visual impairment, which usually affected health-related lifestyles (such as participations in social, physical, and cognitive-stimulating activities). However, these health-related lifestyles were well-recognized beneficial factors of MCI [1, 14]. A cross-sectional study in Japan showed that the elderly with mild visual impairments had more than 2 times higher risk of developing dementia [25]. In contrast, cataract surgery may help visual-impaired patients recover eyesight and lower significantly risks of MCI [25].

Based on the Bayesian network model of causal inference (Figure 1), there are three upstream influencers of bowel movement, namely fruit consumption, hypertension, and cataract/glaucoma. Because these three factors converge on the same node of bowel movement, no direct causal path could be inferred. Commonly consumed fruits could promote a healthy microbiota ecosystem by the actions of their fiber and polyphenolics. This conclusion was supported by a recent randomized controlled trial that consumption of fresh mango (a common tropical fruit in Singapore) significantly improved constipation status (frequency of bowel movements, stool consistency and shape, and duration of constipation) in chronically constipated adults [26]. Although bowel movement was associated with fruit consumption (Supplementary Table 1 and Figure 1), the multivariate logistic regression study confirmed their independent associations to MCI occurrence (Table 3). Here we did not find an association of green vegetable consumption with MCI occurrence, probably because the Southeast Asian residents maintain a good dietary habit of consuming green vegetables regularly. In our study, up to 89.2% of the elders ate green vegetables regularly no less than 4 days per week. Thus, it may be advisable for elders to consume fruits more frequently or daily to promote bowel movement and further prevent cognitive impairments.

We found that the elders with inactive bowel movement (≤3 days per week) had a higher proportion (57.4%) of hypertension comorbidity, compared to the peers with active bowel movement (46.9%, *P*=0.024, Supplementary Table 1). Whether there is a biological causal association directing from hypertension to bowel movement is yet unclear. However, recent studies suggested that the first-line antihypertensive drugs (e.g. diuretics, beta blockers, calcium channel blockers, and angiotensin-converting enzyme/angiotensin receptor blockers) were associated with an increased likelihood of constipation [27, 28].

Finally, a hypothesized connection between cataracts/glaucoma and inactive bowel movement was also suggested by the Bayesian network model of causal inference (Figure 1). The biological connection between these two factors has not been reported in the literature. We speculate that the hypothesized connection might be attributable to behavior or dietary changes among those elders affected by cataracts/glaucoma. Due to compromised vision, the affected elders may experience physical inactivity, which could lead to slower bowel movement. In addition, they may choose to prepare simple dish or have fast food with insufficient fiber consumption, another cause of inactive bowel movement. But these behavior/dietary connections warrant further examination in future cohort studies.

### Limitations

Our study has several limitations. First, it was a cross-sectional study, and the causal relationship of bowel movement with MCI could not be determined with certainty. Therefore, future follow-up studies or longitudinal cohort studies should be conducted to validate the causal relationship. Second, the data (such as lifestyle, dietary habits, and bowel movement) were collected based on self-reporting methods, which might lead to recall bias. To minimize this bias, we excluded all dementia patients in the study. Finally, the present study lacked objective measurements of bowel movement and laboratory examination of stool microbiota. Future studies with constipation clinical diagnoses and the relevant measurements/surveys are needed to improve our results.

## CONCLUSION

In summary, this study demonstrated that active bowel movement and fruit consumption were significantly associated with decreased risks of MCI in the elderly; while medical comorbidities including stroke, hypertension and cataract/glaucoma were positively associated with MCI. The Bayesian network model of causal inference further revealed five hypothesized causal-association paths leading to MCI, namely stroke, bowel movement, years of schooling via fruit consumption, hypertension via stroke and hypertension via cataracts/glaucoma. Particularly, the combination of stroke and inactive bowel movement reached a maximum conditional probability of 60.00%. Therefore, it would be advisable to decrease risks of MCI by adopting a healthy fruit consumption habit, improving bowel movement and by well management of stroke and hypertension.

## MATERIALS AND METHODS

### Ethics

The study was approved by the National University of Singapore Institutional Review Board (No. NUS-IRB-Ref 10-517), and we received informed consent from all individuals who agreed to participate in the study.

### Participants and procedures

This population-based cross-sectional study was conducted from Jul 2011 to Dec 2016. The study population came from the Diet and Healthy Aging Study (DaHA), a community-based longitudinal cohort study that recruited elderly participants (aged ≥60) from a western district of Singapore [29–31]. Well-trained nurses recruited participants via door-to-door census in the district. A demographic questionnaire, the Singapore Modified version of Mini-Mental State Examination (MMSE), the Montreal Cognitive Assessment (MoCA), and a battery of neuropsychological tests were completed by trained research staffs [29, 31]. The inclusive criteria for this study were: 1) Singaporean or permanent residents who live in the West Region of Singapore and 2) aged ≥60. Those who are unable to conduct meaningful face-to-face interviews were excluded. For the current analysis, we further excluded all individuals with a diagnosis of dementia. Finally, a total of 751 respondents were included in the study, of whom 119 were diagnosed with MCI and the remaining 632 individuals without MCI were defined as the cognitive-healthy group.

### Variables

We collected socio-demographic variables (age, gender, education, and marital status), lifestyle and habits (drinking and smoking status), chronic disease status (hypertension, diabetes mellitus, hyperlipidemia, stroke, cataracts/glaucoma, and gastrointestinal problems), psychosocial variables (anxiety disorder), dietary habits (consumption of meat, milk, green vegetables, fruits, legumes, eggs, tofu, seaweed, sea fish, nuts, curry, walnut, tea, and coffee), and bowel movement status (assessed by asking “How often per week do you have a bowel movement within 10 minutes?”) using structured questionnaires at the participants’ homes. During analysis, the responses of bowel movement status were condensed into the two categories of “≤3 per week” and “≥4 per week.” based on the “Rome III Diagnostic Criteria for Functional Constipation”.

### Neurocognitive assessment and diagnosis

The MMSE and MoCA were used to assess cognitive impairment of participants. The summed scores of MMSE ranged from 0 to 30, with higher scores indicating better cognitive performance [31, 32]. Participants with MMSE total scores below the education-specific cut-off values (≤25 for subjects without formal education, ≤27 for primary school education level, and ≤29 for secondary school and above) might have been suffering cognitive impairment and thus needed further assessment [31]. In addition, MoCA is scored on a 30-point scale, where higher scores correspond to better cognitive status [31, 32]. MCI was defined according to Petersen’s criteria [3, 33]. The diagnosis of MCI was made via consensus of a team consisting of a psychiatric epidemiologist, two senior consultant psychiatrists, and other clinical evaluation specialists [34]. As in our previous studies, cognitive impairment was evaluated by locally normed neuropsychological tests, including the Block Design, Digit Span, Rey Auditory Verbal Learning Test, Semantic Fluency, and Color Trails Test, and all MCI patients also met the criteria of mild neurocognitive disorder in the Diagnostic and Statistical Manual of Mental Disorders (DSM) version 5 [34].

### Statistical analyses

Frequencies and percentages were used for categorical variables, while means and standard deviations (SD) were used to describe continuous variables. The comparisons of categorical variables between MCI and non-MCI group were performed using *chi*-square tests, while continuous variables (age, years of schooling) were compared with ANOVA using Statistic Package for Social Science (SPSS) 22.0. A two-sided *P*-value <0.05 was considered statistically significant. The factors with significant or moderate differences under the chi*-*square test and ANOVA (*P*<0.1) were then included in a multivariate logistic regression model.

After identifying the significant factors underlying the status of MCI, a Bayesian network model, structural equation model (SEM), and directed acyclic graph (DAG) approach were integrated to reveal the structure of hypothesized causal associations between significant factors and MCI Meanwhile, the probability inferences of the Bayesian network for the influencing factors of MCI were calculated, and the importance of each significant factor was quantitatively measured based on the Tree Augmented Naïve (TAN) Bayesian network learning. In general terms, the Bayesian network model was fitted with the bnlearn package via R software 3.5, the structure of the Bayesian network was defined using the hill climbing algorithm, and the BIC indicator was used to optimize and evaluate the fitted model. Moreover, the Bayesian-network-based causal relationships between exposure variables and MCI were visualized using the R package “dagitty.” In addition, the metrics of importance for exposure variables were calculated using SPSS Modeler 18.0.

## Supplementary Material

Supplementary Tables
