# Supplementary material for: Inactive bowel movement and stroke are associated with increased risks of mild cognitive impairment among community-living Singapore elderly
Source: Aging (Albany NY). 2020 Sep 9;12(17):17257–70. doi: 10.18632/aging.103674 (PMC7521501; doi:10.18632/aging.103674)
Supplement: Supplementary Tables [file aging-12-103674-s001..pdf]

SUPPLEMENTARY TABLES

Supplementary Table 1. The associations of dietary habits and medical comorbidities with bowel movement.

Variables	Have a bowel movement within 10 minutes (≤3 per week) N=141 N (%)	Have a bowel movement within 10 minutes (≥4 per week) N=610 N (%)	χ^2	P-value
**How often consume meat and meat product**			0.063	0.802
≤3 times per week	77 (54.6)	326 (53.4)		
≥4 times per week	64 (45.4)	284 (46.6)		
**How often consume milk and dairy product**			0.324	0.569
≤3 times per week	73 (51.8)	332 (54.4)		
≥4 times per week	68 (48.2)	278 (45.6)		
**How often consume green vegetables**			1.305	0.253
≤3 times per week	19 (13.5)	62 (10.2)		
≥4 times per week	122 (86.5)	548 (89.8)		
**How often consume fruits**			4.537	**0.033**
≤3 times per week	40 (28.4)	123 (20.2)		
≥4 times per week	101 (71.6)	390 (79.8)		
**How often consume legumes**			0.344	0.558
≤3 times per week	124 (87.9)	525 (86.1)		
≥4 times per week	17 (12.1)	85 (13.9)		
**How often consume eggs**			0.033	0.856
≤3 per week	117 (83.0)	510 (83.6)		
≥4 per week	24 (17.0)	100 (16.4)		
**How often consume coffee**			0.719	0.397
≤3 times per week	39 (27.6)	191 (31.3)		
≥4 times per week	102 (72.4)	419 (68.7)		
**How often consume nuts**			0.647	0.421
≤3 times per week	130 (92.2)	548 (90.0)		
≥4 times per week	11 (7.8)	61 (10.0)		
**How often consume sea fish**			0.006	0.936
≤3 times per week	73 (51.8)	312 (51.4)		
≥4 times per week	68 (48.2)	295 (48.6)		
**How often consume tofu**				
≤3 times per week	123 (87.2)	522 (85.8)	0.182	0.670
≥4 times per week	18 (12.8)	86 (14.2)		
**How often consume carrot**				
≤3 times per week	105 (75.0)	456 (75.0)	< 0.001	1.000
≥7 times per week	35 (25.0)	152 (25.0)		
**How often consume celery**			0.129	0.719
≤3 times per week	132 (93.6)	573 (94.4)		
≥4 times per week	9 (6.4)	34 (5.6)		
**How often consume walnut**			0.206	0.650
≤3 times per week	133 (94.3)	569 (93.3)		
≥4 times per week	8 (5.7)	41 (6.7)		
**How often consume curry**			2.528	0.112
≤3 times per week	134 (95.0)	595 (97.5)		
≥4 times per week	7 (5.0)	15 (2.5)		
**Hypertension**			**5.113**	**0.024**
Yes	81 (57.4)	286 (46.9)		

No	60 (42.6)	324 (53.1)		
**Hyperlipidemia**			2.974	0.396
Yes	83 (58.9)	314 (51.5)		
No	58 (41.1)	296 (48.5)		
**Diabetes mellitus**			0.624	0.430
Yes	28 (19.9)	104 (17.0)		
No	113 (80.1)	506 (83.0)		
**Stroke**			0.327	0.586
Yes	6 (4.3)	20 (3.3)		
No	135 (95.7)	590 (96.7)		
**Cataracts/glaucoma**			0.391	0.532
Yes	46 (32.6)	216 (35.4)		
No	95 (67.4)	394 (64.6)		
**Gastrointestinal problems**			0.776	0.687
Yes	10 (7.1)	56 (9.2)		
No	131 (92.9)	554 (90.8)		
**Anxiety disorder**			4.160	0.125
Yes	4 (2.8)	6 (1.0)		
No	137 (97.2)	604 (99.0)		
**Heart attack**			0.569	0.451
Yes	7 (4.7)	22 (3.6)		
No	134 (95.3)	588 (96.4)		
**Irregular heartbeat**			0.211	0.646
Yes	7 (4.7)	25 (4.1)		
No	134 (95.3)	585 (95.9)		
**Heart failure**			0.529	0.467
Yes	3 (2.1)	8 (1.3)		
No	138 (97.9)	602 (98.7)		

Supplementary Table 2. Inference probabilities of Bayesian network for years of schooling and fruit consumption (%).

Paternal nodes		MCI
**Years of schooling**	**Fruit consumption**	**Yes (%)**
≤5	≤3 per week	32.58
≤5	≥4 per week	22.36
6-11	≤3 per week	20.81
6-11	≥4 per week	17.78
≥12	≤3 per week	18.79
≥12	≥4 per week	17.76

Abbreviations: MCI, mild cognitive impairment.
